# Wrong Place at the Wrong Time

**DOI:** 10.4269/ajtmh.21-0429

**Published:** 2021-09-13

**Authors:** Anil Kallesh, Shalini Gu

**Affiliations:** ^1^ Department of Neonatology, Sarji Hospital, Shivamogga, Karnataka, India;; ^2^ Belgaum Institute of Medical Sciences, Department of Pediatrics, Belgaum, Karnataka, India

It was June 2019, my second summer in Delhi. I had no clue that it would be one of the most memorable days of my life—a day to cherish, even to mark the date on the calendar and celebrate yearly. The whole country was agitated because of a mob attack on a resident doctor in Kolkata, leaving him gravely injured. The whole medical fraternity was gearing up for nationwide protests. I started my day as usual by reporting to the neonatal intensive care unit (NICU) at 8 am sharp. I took charge of the neonates from my very tired fellow resident, as he finished his 24-hour shift.

Amid a whole lot of babies, I stopped beside a sick neonate who needed all my attention. A new challenge waited for me: all the alarms were beeping and the infusion pump lights were flashing. The neonate I was standing in front of was in cardiogenic shock and on ventilatory support with a saturation of 45%. I felt terrible. I thought to myself, *this baby is in the wrong place at the wrong time*.

*How can I feel this way?* I thought. There I was, thinking about a patient who was in an intensive care unit in a hospital in the heart of the capital city of the country. How could this be the wrong place?

This government medical college hospital records more than 15,000 births a year—enough to keep you busy during a 15-hour day shift, with more than 3,000 NICU admissions. Such large, populated hospitals are not uncommon in every part of India. This particular hospital is one of the best and most sought after maternal and child health hospitals in Delhi. It is sought out by patients for treatment, and by students after receiving their bachelor of medicine and bachelor of surgery degrees to receive postgraduation training in pediatrics, obstetrics, and gynecology. The hospital contains many esteemed specialties, such as neonatology, pediatric neurology, pediatric nephrology, pediatric surgery, and pulmonology. The experts available share their years of expertise and experience to ignite our mind and treat the diseased. Despite all this, the doors of this temple of pediatrics are closed when it comes to kids suffering from critical congenital heart disease (CCHD).

Whenever I encounter kids with congenital heart disease, I ask myself, *why is this kid in the wrong place at the wrong time?* There is no pediatric cardiologist nor is there a cardiothoracic vascular surgeon available here to heal the suffering of these children. Instead, we have to refer children to facilities that are already overburdened with long waiting lists. Most children do not survive to their surgery date. Many referrals to such centers are rejected because of a shortage of beds or because the patient is too unstable to transfer. This is why I thought “wrong place at the wrong time.”

Just 5 weeks before, I had returned from 2 months of an “observership” in neonatology at a university hospital in one of the most developed Scandinavian countries as a part of a university exchange program for residents and nurses. I had previously thought of my hospital as very equipped. We are neither stuck in the dense jungles with no roads for an ambulance, nor are we stuck in the Himalayan mountains, covered with snow, waiting for helicopters to fly us and our patients to safety. But, we are not equipped to meet the needs of our kids with CCHD.

Approximately 7 of every 1,000 live-born babies suffer from a variety of congenital heart diseases and 25% of these infants have CCHD. The word *critical* in CCHD means that, without surgical intervention, survival of these babies is impossible. With 15,000 births a year at my hospital, we see approximately 100 newborns with congenital heart disease a year, 25 of whom have CCHD. All such patients in whom we suspect heart disease require echocardiography by a cardiologist. After the cardiologist confirms congenital heart disease, the cardiothoracic vascular surgeon operates on the working heart to correct the defect. This is considered one of the most challenging surgeries in the field of medicine.

During my stay in the Scandinavian university hospital, I saw many such CCHD babies flown in by air ambulance from various parts of the country to the university hospital. It was reassuring that every such baby reached the right place at right time and was operated on. Smiling families walked out of the hospital with their child in their arms. I thought, *when will the kids in my country experience such a marvelous health-care facility?*

In June 2019, as I stood in front of the baby in the NICU, I only wished I could change her course. I thought of our standard practices. I thought of how the cardiologist needed to review the echocardiography. I thought of the risks associated with transport without the security of an air ambulance. And then I thought to myself, *everything changes from changing oneself*. I recorded a video clip of the infant’s echocardiogram and sent the video via WhatsApp messenger to a cardiologist, who interpreted the image as transposition of the great arteries with an intact ventricular septum. The baby was already on mechanical ventilatory support, a prostaglandin infusion, and dopamine and dobutamine infusions.

We contacted the pediatric cardiologists at one of the apex institutes in our country that happen to be located in Delhi, and asked for a balloon atrial septostomy. This is actually only a temporary procedure used to keep infants alive until they can undergo definitive surgery (in this case, arterial switch repair).

Knowing the difficulty of transport, but the urgent need for surgery, the pediatric cardiology team agreed to treat the baby based on my WhatsApp video-based diagnosis of transposition of the great arteries with intact ventricular septum. However, they could not admit her because they lacked the staff to do so; the resident doctors were on strike. They did agree to treat the infant as long as we could return her to the originating hospital after the procedure. It was already past 7 pm. We assembled a transport team comprised of myself, a reliable junior resident, and a transportation kit. We transported the baby on manual ventilation. She underwent the balloon atrial septostomy successfully and we brought her back to our hospital. En route to our hospital, I compressed the angiography site to prevent bleeding. On arrival, the baby was in stable condition and was put back on the mechanical ventilator.

I signed off to the on-duty resident, but the whole night I couldn’t sleep a minute. How could I after such an eventful day?

A surprise was waiting for me the next morning when I signed into the ward. The baby had improved hemodynamically. The blood gas and saturation on the pulse oximeter read 84%, and we were able to taper and wean her from respiratory support. We discharged the patient and connected the parents with a cardiothoracic and vascular surgery center with the help of nongovernment organization funding.

It turns out, for this baby, it was the right place at the right time.

We subsequently lost the patient to follow-up because of the COVID-19 pandemic. However, 1.5 years later, the father brought the child to our clinic. The little girl jumped and played throughout the outpatient department, spreading her smile. Our eyes filled with tears, and all the memories from June 2019 rushed back to us. For an intensivist, there is no greater satisfaction than seeing the most critically ill patient from the NICU come back, smiling, to our follow-up clinic. I urge my fellow doctors who encounter such cases—when you feel your patients are in the wrong place at the wrong time—never to forget these patients may be in hands of those who can create the right place at right time.

